# Metformin Adjunct With Antineoplastic Agents for the Treatment of Lung Cancer: A Meta-Analysis of Randomized Controlled Trials and Observational Cohort Studies

**DOI:** 10.3389/fphar.2021.639016

**Published:** 2021-06-03

**Authors:** Xiaofeng Luo, Xi Chen, Lin Wang, Bowen Yang, Shuang Cai

**Affiliations:** ^1^ Department of Pharmacy, The First Hospital of China Medical University, Shenyang, China; ^2^ School of Pharmacy, China Medical University, Shenyang, China; ^3^ Department of Medical Oncology, The First Hospital of China Medical University, Shenyang, China

**Keywords:** metformin, chemotherapeutic drugs, lung cancer, survival, prognosis, meta-analysis

## Abstract

**Objective:** Resistance to anticancer agents ensures a poor prognosis in patients with lung cancer. Metformin could enhance the anticancer effects of standard antineoplastic agents [traditional chemotherapy drugs, epidermal growth factor receptor tyrosine kinase inhibitors (EGFR-TKIs), or immune checkpoint inhibitors (ICIs)]; however, it is unclear whether metformin can be combined with antineoplastic agents in the treatment of lung cancer. To explore the efficacy of combinational strategies, we performed a systematic review and meta-analysis for diabetic and non-diabetic patients with lung cancer.

**Method:** An electronic literature search was performed to obtain relevant randomized controlled trials (RCTs) and observational cohort studies. Hazard ratios (HR) with 95% confidence intervals (CI) of overall survival (OS) and progression-free survival (PFS) outcomes were extracted. Subgroup analysis by antineoplastic agents, study type, histology and clinical stage were investigated.

**Results:** 14 studies (three RCTs and eleven observational cohort studies) consisting 3,856 patients were included in the meta-analysis. Compared to standard antineoplastic agents alone (traditional chemotherapy drugs, EGFR-TKIs or ICIs), the antineoplastic agents combined with metformin significantly improved OS (HR 0.73, 95% CI 0.66–0.81, *p* < 0.00001) and PFS (HR 0.72, 95% CI 0.59–0.88, *p* = 0.001); a similar association was found in observational evidence. Limited data from RCTs showed no differences in OS or PFS.

**Conclusion:** Metformin plus antineoplastic agents may improve survival outcomes of patients with lung cancer. Further investigation is needed.

## Introduction

Lung cancer is one of the most prevalent cancers, with 2.1 million newly diagnosed cases worldwide in 2018. Lung cancer remains the leading cause of cancer-related mortality, and is responsible for nearly 18% of all cancer deaths (Bray et al., 2018). Most lung cancer patients already have advanced or metastatic lesions at the time of the primary diagnosis, and receive comprehensive chemotherapy-based treatment (Walters et al., 2013). Despite advances in treatment approaches over the past few decades, the 5-years overall survival rate of lung cancer remains poor, at only 19% (Siegel et al., 2020). Moreover, resistance to antineoplastic agents may result in poor prognosis (Ailles et al., 2007; Gazdar, 2009; Restifo et al., 2016). Strategies to sensitize patients to anticancer agents and enhance efficacy are therefore required as well as to repositioning known drugs for cancer treatment, with the advantages of safety and low cost.

Metformin, a commonly used oral antidiabetic drug, has shown potential anticancer properties in epidemiologic studies that found a reduced risk of cancer in patients with diabetes who received metformin compared to those who were treated with other antidiabetic drugs (Zhang et al., 2014). Metformin is known to inhibit cell proliferation and tumor growth primarily by activating adenosine monophosphate-activated protein kinase (AMPK) (Ben Sahra et al., 2011). Subsequently, metformin has been investigated as an antitumor agent for various cancers, including lung cancer (Zhang and Li, 2014; Coyle et al., 2016). Recently, meta-analyses have suggested that metformin intake may be associated with a survival benefit among patients with lung cancer and concurrent diabetes (Cao et al., 2017; Zhang et al., 2018; Xiao et al., 2020). In addition, clinical studies have identified combinational therapies of metformin and antineoplastic agents [traditional chemotherapy drugs, epidermal growth factor receptor tyrosine kinase inhibitors (EGFR-TKIs), or immune checkpoint inhibitors (ICIs)] in lung cancer, both in diabetic and non-diabetic subjects. The results of some studies showed that metformin, when used in combination with antineoplastic agents, appears to improve the antitumor effect of anticancer agents (Tan et al., 2011; Chen et al., 2015; Kong et al., 2015; Lin et al., 2015; Xu et al., 2015; Afzal et al., 2019; Arrieta et al., 2019; Hung et al., 2019). However, several studies have reported no effect of adjuvant metformin therapy on survival outcomes (Ahmed et al., 2015; Sayed et al., 2015; Wink et al., 2016; Lu et al., 2018; Wen-Xiu et al., 2018; Li et al., 2019). Moreover, no systematic review of previous studies has evaluated the efficacy of metformin in combination with anticancer drugs for the treatment of lung cancer.

Therefore, we conducted this meta-analysis to explore the combinational effects of metformin with antineoplastic agents in patients with lung cancer.

## Methods

### Search Strategy

Databases, including PubMed, Embase, the Cochrane Library, and Web of Science, were searched for relevant studies published from 1980 to November 2020 in compliance with the Preferred Reporting Items for Systematic Reviews and Meta-analyses guidelines. The following MeSH terms, together with related text words and keywords, were used for the literature search: metformin AND (lung neoplasms OR lung tumor).

### Study Selection

Studies were selected based on the following inclusion criteria: 1) study design: observational studies or randomized controlled trials (RCTs); 2) subjects: patients with pathologically diagnosed lung cancer; 3) exposure intervention: the experimental group was treated with metformin plus antineoplastic agents, whereas the control group with standard antineoplastic agents alone; 4) outcome indicator: the primary outcome measure was overall survival (OS), and progression-free survival (PFS) was the second outcome measure; and 5) effect estimates of hazard ratio (HR) and corresponding 95% (CI) were directly reported or could be calculated from sufficient data.

Studies were excluded if they met the following exclusion criteria: 1) abstract-only publications; 2) when multiple publications for the same study were found, papers with the most comprehensive or most recent data were used; 3) treatment strategy was unclear; 4) insufficient data/information was provided, and 5) studies that were not published in English.

### Data Extraction and Quality Assessment

Two reviewers independently extracted the following information from each of the included studies: author, publication year, region, study period, study design, sample size, age, comparison with basic population, anticancer treatment, lung cancer histology, stage, median follow-up time, diabetes status, and HR with the corresponding 95% CI of outcome measures. The quality assessment of the included RCTs and observational studies was rated using the Jadad Scale and Newcastle–Ottawa scale (NOS) scoring methods, respectively. Discrepancies in data extraction and quality assessment were resolved through a discussion with a third reviewer.

### Statistical Analysis

Data were processed using Review Manager 5.3 software (The Cochrane Collaboration, Oxford, United Kingdom). The HRs with the corresponding 95% CI of each study were used to calculate the pooled effect of combination therapy with metformin and anticancer drugs on lung cancer. Statistical heterogeneity was calculated using Higgins’s *I* ^*2*^ test: I^2^ > 50% indicated substantial heterogeneity, and a random-effect model was used; otherwise, a fixed-effect model was applied. Subgroup analysis was conducted based on the antineoplastic agents, study type and histology. Publication bias was assessed with a funnel plot. Sensitivity analysis was performed by sequential omission of each study to examine the robustness of the pooled results.

## Result

### Search Results

**FIGURE 1:**
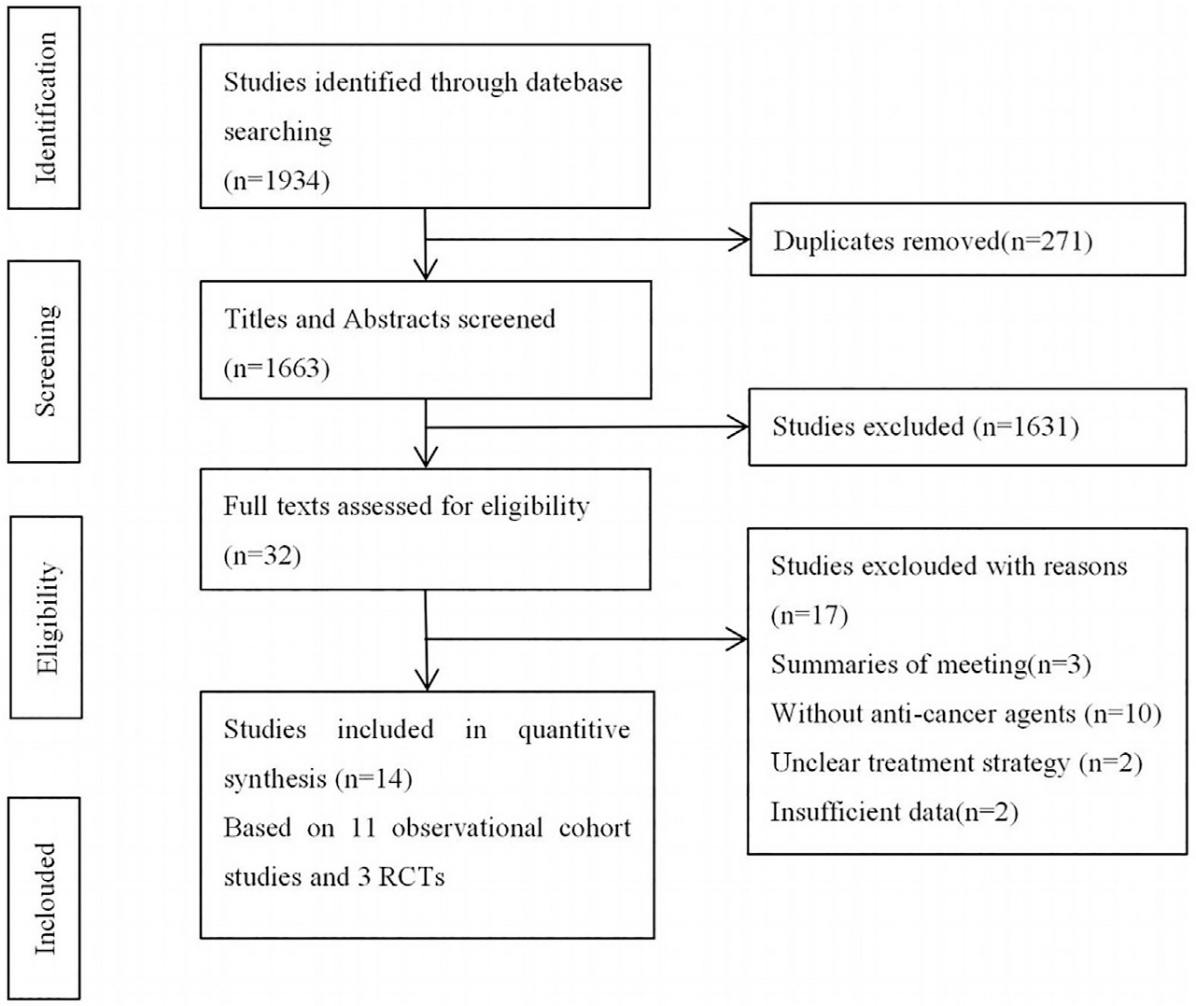
Flowchart of study selection.

Initially, we identified 1,934 studies from the electronic database searches. Among these, duplicates and irrelevant studies or those without sufficient data were excluded, and 14 studies were enrolled in this systematic review (Figure 1), comprising 11 cohort studies and 3 RCTs.

### Study Characteristics and Quality Evaluation

Table 1 shows the main characteristics of the included studies, which were all published in the recent decade (2011–2019). A total of 1,202 patients received metformin in combination with antineoplastic agents (traditional chemotherapy drugs, EGFR-TKIs, or ICIs), and 2,654 patients received antineoplastic agents alone. Nine of the included studies focused on subjects receiving antineoplastic agents with traditional chemotherapy drugs, four of the studies with EGFR-TKIs, one with ICIs.

**TABLE 1:** Main characteristics of included studies.

Author, year	Study region	Study design	Histology	Stage	Treatment strategy	Sample size	Median fellow-up (months)	DM
Tan et al. (2011)	China	Cohort	NSCLC	Ⅱ-Ⅳ	Chemotherapy	99	NR	Yes
Ahmed et al. (2015)	United States	Cohort	NSCLC	Ⅰ-Ⅳ	Chemoradiotherapy	40	17.0	Yes
Lin et al. (2015)	United States	Cohort	NSCLC	Ⅳ	Chemotherapy	349	NR	Yes
Chen et al. (2015)	China	Cohort	NSCLC	Ⅲ-Ⅳ	EGFR-TKI	90	NR	Yes
Kong et al. (2015)	China	Cohort	SCLC	Limited and extensive	Chemotherapy	259	68.0	Yes
Xu et al. (2015)	China	Cohort	SCLC	Limited and extensive	Chemotherapy	79	65.0	Yes
Sayed et al. (2015)	Egypt	RCT	NSCLC	Ⅳ	Chemotherapy	30	NR	No
Wink et al. (2016)	Netherlands	Cohort	NSCLC	Ⅱ-Ⅲ	Chemoradiotherapy	682	30.0	Yes
Wen-Xiu et al. (2018)	China	Cohort	NSCLC	Ⅰ-Ⅳ	Chemotherapy	75	58.7	Yes
Lu et al. (2018)	China	Cohort	SCLC	Limited and extensive	Chemotherapy	32	NR	Yes
Hung et al. (2019)	China	Cohort	NSCLC	Ⅲ-Ⅳ	EGFR-TKI	1,633	21.5	Yes
Afzal et al. (2019)	United States	Cohort	NSCLC	Ⅳ	ICIs	50	NR	Mixed
Li et al. (2019)	China	RCT	NSCLC	Ⅲ-Ⅳ	EGFR-TKIs	224	19.2	No
Arrieta et al. (2019)	Mexico	RCT	NSCLC	Ⅲ-Ⅳ	EGFR-TKIs	139	16.9	No

*NR*, not reported; *NSCLC*, non-small cell lung cancer; *SCLC*, small cell lung cancer; *EGFR-TKIs*, epidermal growthfactor receptor-tyrosine kinase inhibitors; *ICIs*, immune checkpoint inhibitors; *RCT*, randomized controlled trial; *DM*, diabetes mellitus.

The results of the quality assessment are summarized in Tables 2, 3. All of the included studies were of high quality, with Jadad scores ranging from four to seven and NOS scores of 7–9.

**TABLE 2:** Quality evaluation of randomized controlled trials (RCTs) included according to Jadad Score.

Study	Randomization present	Allocation concealment	Blinding	Follow up	Total score
Sayed et al. (2015)	2	2	0	1	5
Li et al. (2019)	2	2	2	1	7
Arrieta et al. (2019)	2	1	0	1	4

Total possible scores: 0–7 points, considered poor quality if < 4.

**TABLE 3:** Quality evaluation of observational studies included according to the Newcastle-Ottawa Scale (NOS).

Study	Selection				Comparability	Outcome			Score
	A	B	C	D	E	F	G	H	
Tan et al. (2011)	★	★	★	★	★★	★	★	★	9
Ahmed et al. (2015)	★	★	★	★	★★	★			7
Lin et al. (2015)	★	★	★	★	★★	★	★		8
Chen et al. (2015)	★	★		★	★★	★	★		7
Kong et al. (2015)	★	★		★	★★	★	★	★	8
Xu et al. (2015)	★	★	★	★	★★	★	★	★	9
Wink et al. (2016)	★	★		★	★★	★	★	★	8
Wen-Xiu et al. (2018)	★	★	★	★	★★	★	★	★	9
Lu et al. (2018)	★	★		★	★★	★	★		7
Hung et al. (2019)	★	★	★	★	★★	★	★		8
Afzal et al. (2019)	★	★	★	★	★	★	★	★	8

Total possible scores: 0–9 points, considered poor quality if < 7.

*A*, Representativeness of the exposure cohort; *B*, Selection of the non-exposed cohort; *C*, Determination of exposure factor; *D*, Outcome indicators not present at the beginning of study; *E*, Control for important confounders or additional factors; *F*, Evaluation of outcome indicators; *G*, The follow-up time is long enough; *H*, Adequacy of follow-up cohorts.

### Quantitative Synthesis

The 14 studies were pooled in the meta-analysis. All survival outcomes are shown in Table 4.

**TABLE 4:** Meta-analysis on survival outcomes.

Outcome	Number of studies	Pooled HR (95%CI)	*P* value	Heterogeneity		Analysis model
				*P*_h_	I^2^	
OS	14	0.74 (0.68–0.81)	<0.00001	0.05	42	F
PFS	11	0.81 (0.74–0.88)	<0.00001	0.04	48	F

*OS*, overall survival; *PFS*, progression-free survival; *HR*, hazard ratio; *CI*, confidence interval; *R*, random effect model; *F*, fixed effect model.

#### OS

**FIGURE 2:**
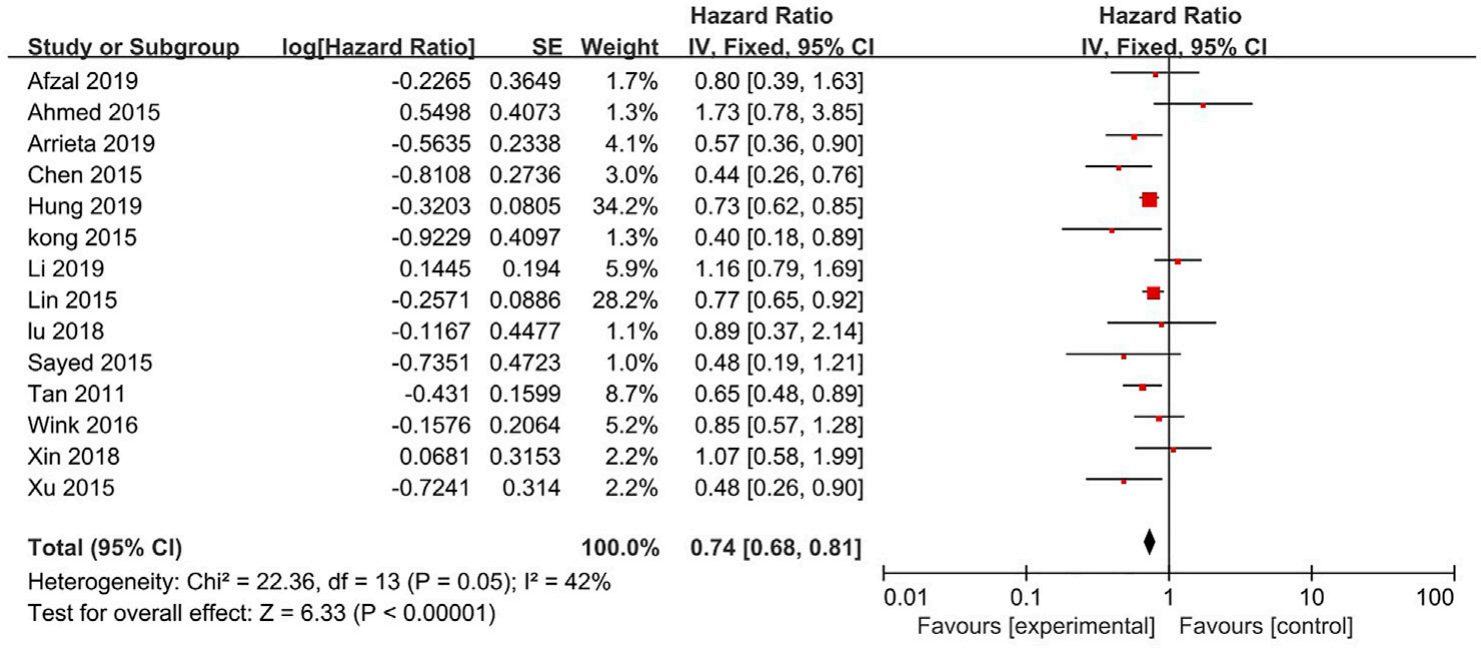
Forest plot for the meta-analysis on overall survival.

14 studies reported the OS (Figure 2). The results indicates that the treatment of metformin plus antineoplastic agents significantly improved the OS of patients with lung cancer compared with the standard antineoplastic agents alone (HR = 0.74, 95% CI = 0.68–0.81; *p* < 0.00001) with a fixed-effect model (*p* = 0.05, I^2^ = 42%).

#### PFS

**FIGURE 3:**
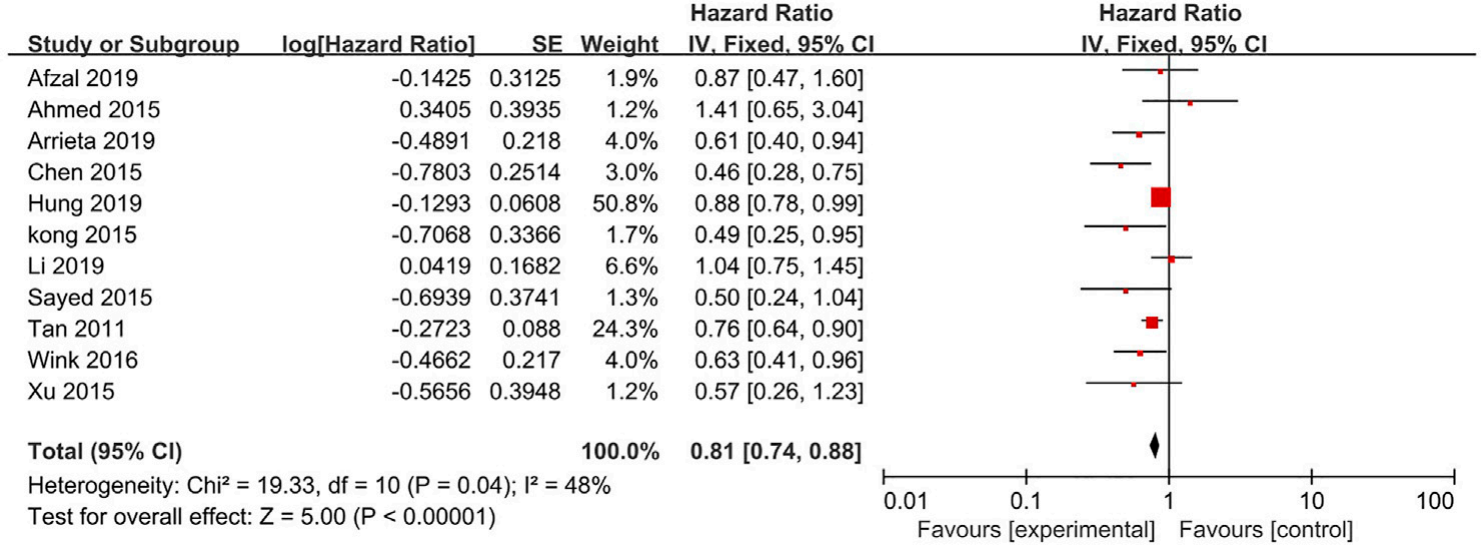
Forest plot for the meta-analysis on progression-free survival.

11 studies reported the PFS (Figure 3). The results showed a significant improvement in the PFS for the metformin combination group (HR = 0.81, 95% CI = 0.74–0.88; *p* < 0.00001) based on analysis in a fixed-effect model (*p* = 0.04, I^2^ = 48%).

### Subgroup Analysis

The outcomes of subgroup analysis are shown in Table 5.

**TABLE 5:** Subgroup analysis of survival outcomes.

Outcome	Subgroup		Number of studies	Pooled HR (95%CI)	*p* Value	Heterogeneity		Analysis model
						*P*_h_	I^2^ (%)	
OS	Antineoplastic agents	Chemotherapy drugs	9	0.75 (0.66–0.86)	<0.0001	0.14	34	F
		EGFR-TKIs	4	0.71 (0.51–0.98)	0.004	0.02	70	R
	Study type	RCTs	3	0.73 (0.41–1.30)	0.29	0.03	71	R
		Cohort studies	11	0.73 (0.67–0.81)	<0.00001	0.13	33	F
	Histology	NSCLC	11	0.75 (0.69–0.83)	<0.00001	0.06	44	F
		SCLC	3	0.53 (0.35–0.81)	0.0003	0.38	0	F
	Stage	Ⅲ–Ⅳ	7	0.74 (0.67–0.82)	<0.00001	0.08	46	F
		Ⅰ–Ⅳ	4	0.89 (0.63–1.27)	0.53	0.1	52	R
PFS	Antineoplastic agents	Chemotherapy drugs	6	0.72 (0.63–0.84)	<0.0001	0.28	20	F
		EGFR-TKIs	4	0.76 (0.57–1.01)	0.06	0.02	70	R
	Study type	RCTs	3	0.73 (0.47–1.15)	0.17	0.06	64	R
		Cohort studies	8	0.81 (0.74–0.88)	<0.00001	0.04	48	F
	Histology	NSCLC	9	0.77 (0.65–0.91)	0.002	0.04	51	R
		SCLC	2	0.52 (0.32–0.86)	0.01	0.79	0	F
	Stage	Ⅲ–Ⅳ	7	0.77 (0.65–0.91)	0.003	0.04	54	R
		Ⅰ–Ⅳ	3	0.76 (0.65–0.89)	0.0006	0.2	38	F

*NSCLC*, non-small cell lung cancer; *SCLC*, small cell lung cancer; *EGFR-TKIs*, epidermal growth factor receptor-tyrosine kinase inhibitors; *HR*, hazard ratio; *CI*, confidence interval; *R*, random effect model; *F*, fixed effect model; *OS*, overall survival; *PFS*, progression-free survival.

#### OS

A subgroup analysis on studies comparing metformin plus antineoplastic agents (chemotherapy drugs and EGFR-TKIs) with standard antineoplastic agents alone was performed, demonstrating a significant survival benefit for the chemotherapy drugs group (HR = 0.75, 95% CI = 0.66–0.86, *p* < 0.0001) and EGFR-TKIs groups (HR = 0.71, 95% CI = 0.51–0.98, *p* = 0.03). The stratification analysis by histology revealed a similar protective effect in both the non-small cell lung cancer (NSCLC) group (HR = 0.75, 95% CI = 0.69–0.83; *p* < 0.00001) and small cell lung cancer (SCLC) group (HR = 0.53, 95% CI = 0.35–0.81; *p* = 0.0003). When stratified by clinical stage, we found survival benefit in patients at stage III–IV treated with metformin (HR = 0.74, 95% CI = 0.67–0.82; *p* < 0.00001), but not the group at stage I–IV (HR = 0.89, 95% CI = 0.63–1.27; *p* = 0.1). Subgroup analysis on cohort studies showed a longer OS for the metformin combined with antineoplastic agents group while that of the RCTs did not demonstrate a statistical significance.

#### PFS

We performed subgroup analyses for PFS by antineoplastic agents, a significant protective effect on PFS was observed in the chemotherapy drugs group (HR = 0.72, 95% CI = 0.63–0.84, *p* < 0.0001) but not EGFR-TKIs groups (HR = 0.76, 95% CI = 0.57–1.01, *p* = 0.02). With regard to histology, the protective effect persisted in the NSCLC group (HR = 0.77, 95% CI = 0.65–0.91; *p* = 0.002) and SCLC groups (HR = 0.52, 95% CI = 0.32–0.86; *p* = 0.01). In the stratification analyses by clinical stage, such survival benefit was also obviously observed in patients at stage III–IV (HR = 0.77, 95% CI = 0.65–0.91; *p* = 0.003) and stage I–IV (HR = 0.76, 95% CI = 0.65–0.89; *p* = 0.0006). Subgroup analysis on cohort studies showed a improved PFS for the metformin plus antineoplastic agents group while that of the RCTs did not demonstrate a statistical significance.

### Publication Bias

**FIGURE 4:**
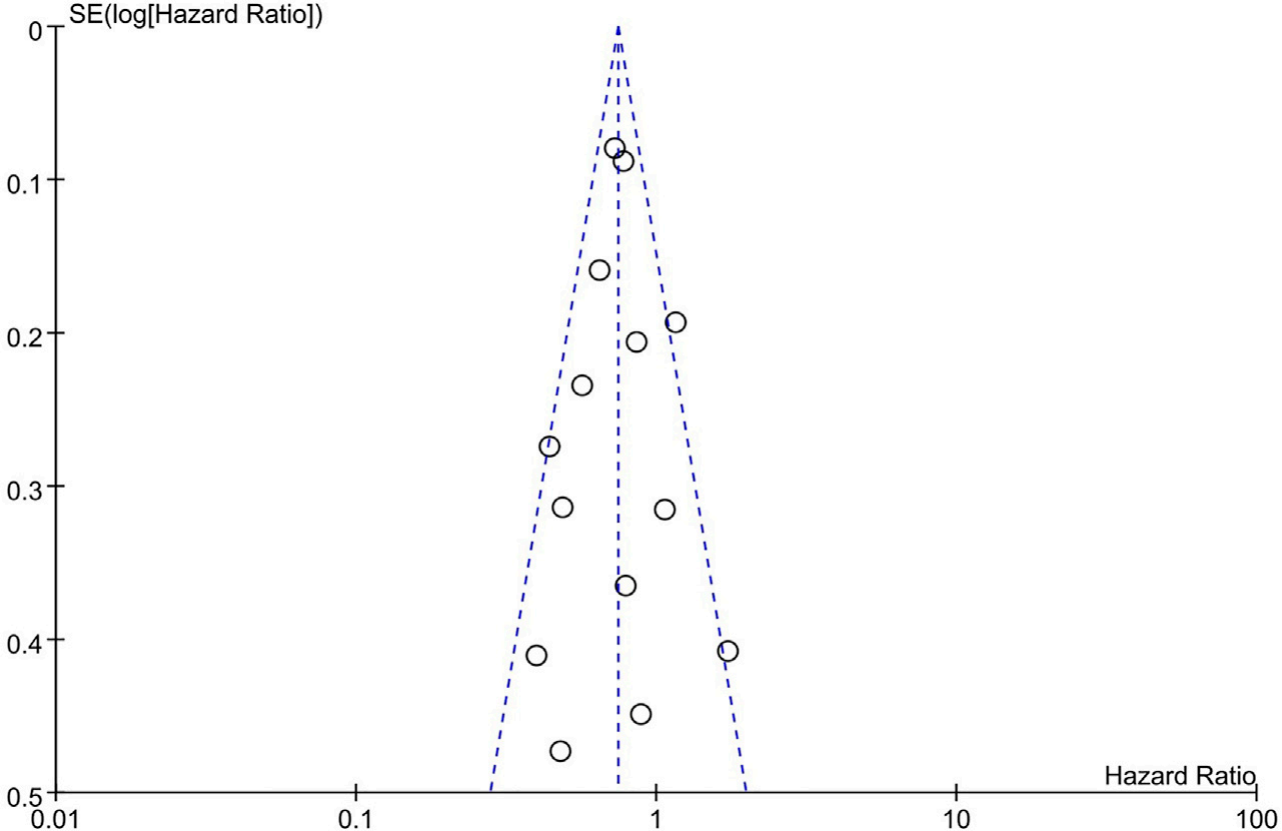
Publication bias assessment of primary outcome.

The funnel plot seemed to be symmetrical (Figure 4), which indicated a slight publication bias of the studies included in this analysis.

### Sensitivity Analysis

No single study markedly altered the overall effect in the sensitivity analysis, suggesting that the pooled HR of OS was stable.

## Discussion

### Summary of the Main Findings

This systematic review and meta-analysis evaluated the combinational efficacy of metformin and antineoplastic agents in patients with lung cancer, including diabetic and non-diabetic subjects. The results of pooled analysis demonstrated that metformin, in combination with antineoplastic agents, may improve the PFS and OS compared to standard antineoplastic agents alone. Specially, the survival benefit may be more evident in patients with advanced NSCLC receiving chemotherapy treatment.

Some mechanisms for the therapeutic effect of metformin on lung cancer have been proposed. Studies have shown that the hyperglycemia and hyperinsulinemia that are associated with diabetes can increase tumor growth (Pollak, 2008; Shlomai et al., 2016). AMPK is a key molecule in the regulation of cellular energy metabolism. Metformin increases the receptor sensitivity to insulin and enhances peripheral glucose uptake by activating the AMPK pathway, which may reduce the action of insulin in promoting tumor growth (Bailey, 2005). In addition, mammalian target of rapamycin (mTOR) is an important regulator of cell growth and proliferation. Studies have shown that abnormal regulation of the mTOR signaling pathway is closely related to cell proliferation (Saxton and Sabatini, 2017). Moreover, activated AMPK can inhibit mTOR, and then induce tumor cell apoptosis, cell-cycle arrest, and autophagy, thereby directly conferring an antitumor effect. In addition, metformin may play a role in combination with anticancer agents. Lin et al. (2013) reported that metformin could augment cisplatin cytotoxicity and help overcome tumor drug resistance by suppressing the signal transducer and the activator of transcription-3 activity. Subsequently, some studies demonstrated that metformin may enhance the efficacy of platinum-based chemotherapy (Teixeira et al., 2013; Moro et al., 2018). In addition, when metformin is added to paclitaxel, there was detectable quantitative potentialization of molecular signaling through the AMPK pathway and promotion of apoptosis through arresting of cells in the G2/M phase (Rocha et al., 2011), which augments the cytotoxic effect induced by paclitaxel (Tseng et al., 2013). Furthermore, metformin sensitizes EGFR-TKI-resistant human lung cancer cells by inhibiting interleukin-6 (IL-6) signaling, reversing epithelial–mesenchymal transition (EMT) (Li et al., 2014), and targeting the insulin-like growth factor-1 receptor (IGF-1R) signaling pathway (Pan et al., 2018). Moreover, remodeling the hypoxic tumor microenvironment and blocking the inhibitory signal of PD-L1 by metformin enhances cytotoxic T lymphocyte (CTL) activity against cancer cells, which increases the efficacy of immunotherapy drugs (Scharping et al., 2017; Cha et al., 2018). Consequently, metformin has the potential to be used in combination with antineoplastic agents in lung cancer.

Whereas, the variation in the adjuvant effects of metformin administration could be partially interrupted by the differences in treatments types, lung cancer subtypes, and clinical stages. Subgroup analysis indicated synergistic effects in the chemotherapy drugs group, which was in agreement with a previous study (Wan et al., 2016). In addition, we found that patients receiving EGFR-TKIs therapy had better OS. With regard to histology, the protective effect persisted in the NSCLC group and SCLC groups. When stratified by clinical stage, such survival benefit by metformin adjuvant treatment was also obviously observed in patients at advanced stage (Ⅲ–Ⅳ) but not in mixed stage (I–IV). Furthermore, metformin did not show benefit in RCTs (which focus on non-diabetic patients), only in observational studies (which investigate patients with concurrent diabetes). However, few studies have investigated the therapeutic effects of metformin for lung cancer at various pathological subtypes, different stages and the distinct role and advantages of metformin therapy in combination with various treatment strategies. Besides, whether metformin will be universally efficacious in lung cancer therapy or more effective in diabetic patients are warranted to be determined.

### Strengths of This Analysis

To our knowledge, this study is the first systematic review and meta-analysis to present the latest clinical evidence with regard to the combinational effects of metformin with anticancer agents on lung cancer survival in both diabetic and non-diabetic subjects. This is the most comprehensive meta-analysis for lung cancer patients, comprising RCTs and observational cohort studies whereas all of the earlier meta-analyses included cohort studies only. In addition, we identified OS and PFS outcome indicators, whereas most of the earlier meta-analyses only observed OS. Furthermore, subgroup analysis was performed for different antineoplastic drug, lung cancer histology, clinical stage and study type. Collectively, The current clinical evidence supported a favor for metformin-assisted antineoplastic chemotherapy in lung cancer patients, particularly in advanced NSCLC receiving traditional chemotherapeutic agents.

### Limitations of This Analysis

The findings of this study need to be cautiously interpreted in light of several limitations. First, the inherent lack of equal probability to allocation to treatment or control (metformin or not) in observational studies and the possibility that non-observed variables determine that allocation, resulting in selection bias. Besides, the risk of time-related bias may have impacts on the effect of metformin in lung cancer. Second, Most subjects of the cohort studies investigated were diabetics patients. Diabetes is also another complex and heterogeneous condition and factors that may be associated with a prescription of metformin or any other anti-diabetic treatment may be associated the differences in lung cancer OS-PFS identified in those studies. Metformin is the first-line therapeutic drug for type 2 diabetes, especially in patients with early-stage disease. Therefore, most non-metformin patients may have advanced diabetes. We assumed that poor glycemic control and serious diabetic complications may result in inferior clinical outcomes in the non-metformin group to some extent. Besides, diabetic cancer patients treated with more hypoglycemic agents are quite universal in clinical practice. Consequently, it is hard to determine the specific clinical significance of metformin therapy without eliminating the potential influence of other hypoglycemic agents. And well-designed RCTs and large prospective studies are warranted to explore the feasibility of the combinational strategies on patients with lung cancer and diabetes. Third, the number of the RCTs we included is small (only 3 eligible for meta-analysis), which may be related to the newer research field. In addition, all of the RCTs focus on non-diabetic patients. It remains unclear whether combinational strategies involving metformin will become available for patients without diabetes, and there is a limitation in the use of metformin in non-diabetic patients because it is constrained by the approved indications, which again prompts more RCTs to assess the clinical significance. Consequently, whether metformin will be universally efficacious in lung cancer therapy or more effective in diabetic patients are warranted to be determined. Fourth, to ensure the quality of the articles, we limited to select papers published only in PubMed, Embase, the Cochrane Library, and Web of Science, and there might be missed some important publications with negative results that were published in low-rating journals. Fifth, there was great variability in the sample size of the included studies. One of the studies included 1,633 patients, which may have caused the results of the study to dominate the results of the meta-analysis. Therefore, we excluded the study with a large sample size and undertook further analyses. The results showed that the combinational strategies still significantly improved the OS (HR 0.75, 95% CI 0.67–0.84, *p* < 0.00001) and the PFS (HR 0.74, 95% CI 0.65–0.83, *p* < 0.00001) in patients with lung cancer. Sixth, this meta-analysis only included a small number of studies, which could limit the evaluation of the survival outcomes. Furthermore, clinical details including metformin dose, age, and the median follow-up time were insufficient in some studies, potentially leading to heterogeneity among studies. Therefore, this condition limited our ability to explore the sources of heterogeneity and made the subgroup analysis less effective and representative, which suggests the need for more studies to evaluate the effectiveness of metformin. Lastly, additional studies are needed to confirm the stage in which metformin therapy is beneficial and the distinct role and advantages of metformin therapy in combination with various treatment strategies.

## Conclusions

Metformin combined with antineoplastic agents may improve OS and PFS in patients with lung cancer. In view of the limitations of this conclusion, well-designed RCTs and large perspective studies are warranted to explore the feasibility of the combinational strategies.

## Data Availability

The original contributions presented in the study are included in the article/Supplementary Material, further inquiries can be directed to the corresponding author.
